# A comparison of stereotactic and tomosynthesis‐guided localisation of impalpable breast lesions

**DOI:** 10.1002/jmrs.348

**Published:** 2019-07-25

**Authors:** Carolyn Madeley, Meredith Kessell, Chris Madeley, Donna Taylor

**Affiliations:** ^1^ Department of Diagnostic and Interventional Radiology Royal Perth Hospital Perth Western Australia Australia; ^2^ Breast Screen Western Australia Perth Western Australia Australia; ^3^ SEA Pty LTD Perth Western Australia Australia; ^4^ Medical School, Faculty of Health and Medical Sciences University of Western Australia Crawley Western Australia Australia

**Keywords:** accuracy, breast, lesion localisation, stereotactic, tomosynthesis

## Abstract

**Introduction:**

Impalpable breast cancers require precise pre‐operative lesion localisation to minimise re‐excision rates. Conventional techniques include hookwire insertion using stereotactic guidance. Newer techniques include the use of tomosynthesis guidance and the use of iodine‐125 seeds. This study compares the accuracy of lesion localisation with hookwire or seed insertion using prone stereotactic or upright tomosynthesis guidance.

**Methods:**

This registered quality improvement activity did not require formal ethics approval. The post‐localisation images for 116 lesions were reviewed. The distance from the lesion or breast biopsy marker to the hookwire or seed was measured on post‐insertion mammograms. The relative placement accuracy of hookwire or seed using prone stereotactic or upright tomosynthesis guidance was compared. A lesion to seed or wire distance > 10 mm was considered technically unsatisfactory.

**Results:**

94.8% of the seeds and wires inserted via prone stereotactic guidance were accurately placed, compared with 89.6% of those inserted via upright tomosynthesis. There were twice as many technically unsatisfactory insertions under upright tomosynthesis guidance. The majority of the unsatisfactory insertions using upright tomosynthesis occurred when the lesion was at or below the level of the nipple and the insertion was performed craniocaudally.

**Conclusion:**

The degree of accuracy of pre‐operative localisation of impalpable breast lesions is significantly higher with the use of prone stereotactic rather than upright tomosynthesis guidance. This was most evident with the placement of I‐125 seeds, and in cases where the target lesion was located below the level of the nipple.

## Introduction

Mammographic screening programmes, combined with improved imaging techniques, have resulted in the identification of a large number of impalpable breast lesions, which require localisation under imaging guidance for successful surgical excision.^1^, ^2^

Historically, the most common method for pre‐operative localisation of impalpable lesions has been via the insertion of a hookwire under ultrasound or mammographic guidance.^3^ More recently, Radioguided Occult Lesion Localisation using Iodine‐125 Seed (ROLLIS) has been shown to be an accurate alternative.^4^

European guidelines indicate a minimum 90% of localising wires should be within 10 mm of the lesion in any plane,^5^ with a target of 95% or above being desirable.^6^ A meta‐analysis by Fusco et al.^7^ found a range of reported successful wire locations of between 65% and 100%. A similar study conducted by Barentsz et al.^8^ the same year found the reported success rate of radioactive seed localisations to be between 92.8% and 100%.

At our institution, pre‐operative localisation, using either a hookwire or a radioactive seed, is performed preferentially under ultrasound guidance if the lesion or accurately placed biopsy marker clip is sonographically visible. Mammographic guidance, using either conventional stereotactic or tomosynthesis control, is undertaken if the lesion/clip cannot be sonographically visualised. Pending discussion with the surgeon, a distance between the hookwire or radioactive seed of greater than 10 mm, is usually considered unacceptable and prompts insertion of a further hookwire.

Procedures performed using tomosynthesis guidance are being increasingly used in clinical practice as they are quicker and easier to perform than those using conventional grid or stereotactic guidance.^9^, ^10^ They have also been shown to be associated with a lower radiation dose, as tomosynthesis procedures typically require a smaller number of exposures to perform.^10^ Whether the accuracy of tomosynthesis‐guided techniques differs from that of conventional techniques is clinically important but has yet to be evaluated.

The purpose of this study was to compare the use of conventional prone stereotactic guidance with upright tomosynthesis guidance for pre‐operative lesion localisation, to determine whether there is any difference in the accuracy of these two methods.

## Materials and Method

This retrospective review was registered as a clinical audit and quality improvement activity (GEKO Quality Activity 17545), not requiring ethics approval. Data were gathered from all patients (*n* = 130) who underwent mammographically guided pre‐operative localisation at our institution between 1 July 2015 and 31 December 2017. Ten patients who required bracketing wires or seeds, three who had no visible residual lesion where the position of the biopsy marker was deemed unacceptable and one for whom post‐wire mammograms were unavailable on the PACS system were excluded, leaving 116 patients in the study.

The localisation procedures were performed by one of five consultant radiologists, or one of 13 radiology fellows or registrars under consultant supervision.

**Figure 1:**
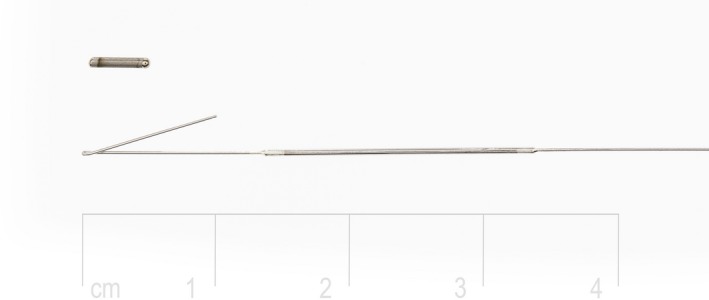
Photograph showing an iodine‐125 seed (length 4.5 mm) and the distal portion of a modified Kopans hookwire. The wire has a 20‐mm‐thickened segment, designed to provide the surgeon with a further visual and tactile guide to the location of the lesion along the length of the wire.

Hookwire insertion was performed using a 20G modified Kopans hookwire, which has a 20‐mm‐thickened segment situated 12 mm from the tip of the hook (Cook Medical, Bloomington, IN, USA; See Fig. 1).

The radiologists aimed to insert the wire through the lesion such that the thickened part of the wire was seen to traverse the lesion in two planes on the post‐wire mammograms, or to lie immediately adjacent to the marker clip should no visible residual lesion remain.

Insertion of a radioactive seed involved placing a small, 4.5 mm × 0.8 mm, titanium seed containing iodine‐125 (ADVANTAGE, IsoAid, Port Richey, FL, USA) (Fig. 1) as close as possible to the centre of the target lesion or immediately adjacent to the marker clip. Pre‐loaded needle kits (Advantage‐Load) were used,^11^ and the seed deployed by fully advancing a stylet within the needle. The seed (and lesion) was then localised in theatre with the use of a hand‐held gamma probe and excised.^12^ The successful excision of the index lesion is confirmed by intra‐operative specimen radiography.

Twenty‐two of the patients included in our study were enrolled in the Radioguided Occult Lesion Localisation using Iodine‐125 Seed (ROLLIS) randomised controlled trial (ACTRN12613000655741) in which participants were randomised to undergo hookwire localisation or ROLLIS. For the remaining patients, the choice between the use of wire or seed was dependent on the availability of localisation booking slots in relation to the date of surgery. Patients who underwent hookwire localisation had their procedure on the day of surgery, while ROLLIS patients had their seed inserted up to eight days prior to surgery.

For stereotactic guided wire or seed localisation, the patient was positioned on a prone table (Lorad, Hologic, Bedford, MA) according to the approach determined by the performing radiologist. A scout image was obtained to confirm the position of the target lesion, and then, a stereotactic pair was taken for targeting purposes. If using a wire, the needle was advanced 10–15 mm beyond the target, to adjust for the position of the thickened part of the wire, while the needle containing the seed was placed at the centre of the lesion. Once the needle was in position, a second stereotactic pair was taken to confirm position prior to deployment.

For tomosynthesis‐guided wire or seed localisation, a Selenia Dimensions system (Hologic) was used. The patient was positioned on a dedicated biopsy chair, either seated or in a lateral decubitus position. The approach taken for localisation, either CC or lateral, was at the discretion of the performing radiologist.^10^ The aim was to traverse the shortest skin to lesion distance possible with the wire wherever possible.^11^, ^13^ The image that most clearly depicted the calcifications, mass, architectural distortion or marker clip was selected, and a crosshair placed on the target.^14^ The images were then scrolled to the skin level, and the X and Y coordinates were determined from the alphanumeric grid markings along the sides of the open biopsy paddle. The depth was determined from the slice number of this image subtracted from the overall number of slices in the tomosynthesis set, as the slices are in 1 mm increments.^14^ Under tomosynthesis guidance, the position of the tip of the needle in relation to the lesion is directly visualised by completing a tomosynthesis sweep, and the position and depth were adjusted prior to deployment, when the radiologist was satisfied with the final position.

Following the localisation procedure, all patients underwent two‐view mammography to document the position of the wire or seed in relation to the marker clip. Positioning was considered acceptable if the seed or thickened part of the wire was within 10 mm of the centre of the lesion or marker clip.^12^

The accuracy of placement of the wire or seed in relation to the lesion was assessed as per Taylor et al.^12^ by measuring the shortest distance (SD) from the seed or thickened segment of the wire to the lesion on the craniocaudal (CC) and lateral (lat) post‐procedural mammograms. If the wire or seed was short of the lesion, this was recorded as a negative value; conversely, if the wire or seed was past the lesion, this was recorded as a positive value.

**Figure 2:**
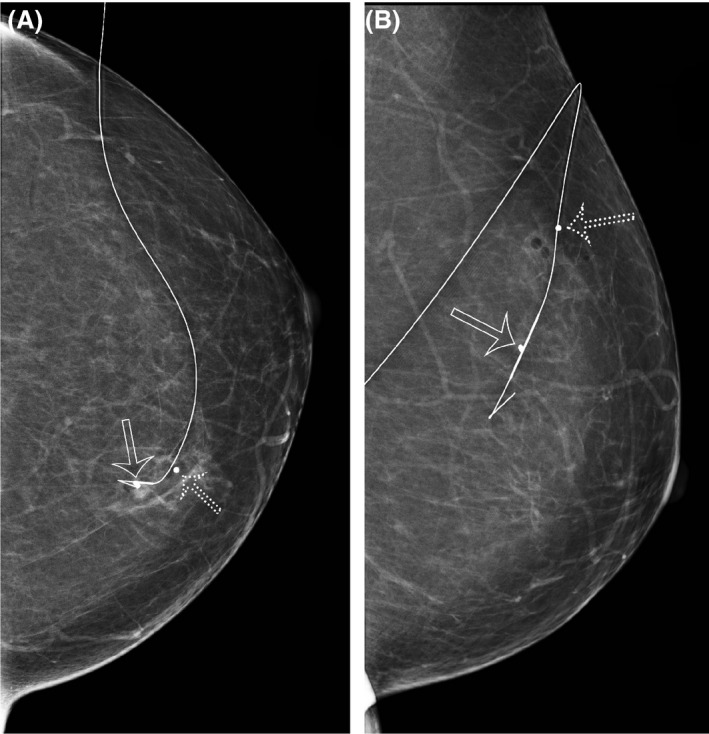
CC (A) and lateral (B) mammograms show optimal wire positioning, with the wire traversing the lesion such that the thickened segment lies at the lesion site. In this case, a biopsy marker is also present within the lesion (hollow arrow). A ball bearing marks the skin entry point (dotted arrow).

**Figure 3:**
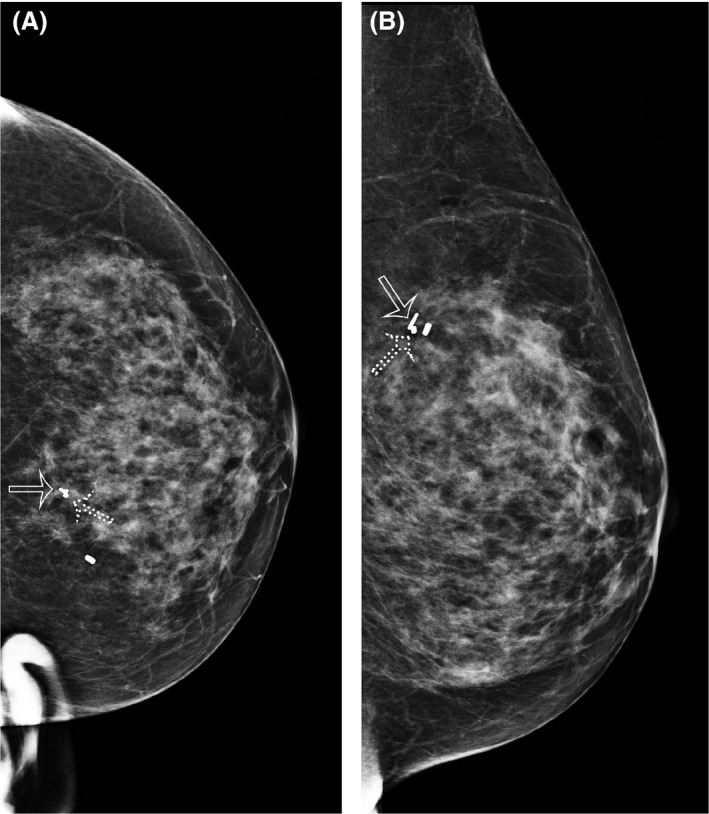
CC (A) and lateral (B) mammograms post‐seed insertion. There are two breast biopsy markers in situ. A dumbbell‐shaped breast biopsy marker (dotted arrow) had been placed at the site of a malignant lesion and was targeted for seed insertion. Positioning here is optimal, with the seed (hollow arrow) lying immediately adjacent to the dumbbell‐shaped breast biopsy marker in two planes.

**Figure 4:**
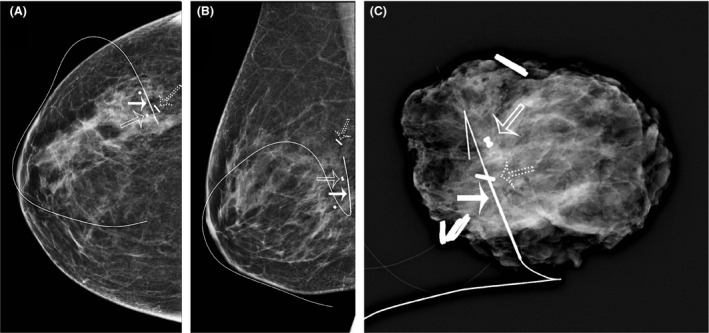
CC (A) and lateral (B) mammograms taken after hookwire insertion to correct an unsatisfactory seed localisation procedure. The dumbbell‐shaped marker clip (hollow arrow) was the localisation target. The SD for the seed (dotted arrow) was 18 mm. Note the optimal position of the wire (solid arrow), with the thickened segment lying immediately adjacent to the biopsy marker. (C) Intra‐operative specimen X‐ray. The specimen has been orientated with medium‐sized Liga clips (one for superior, two for medial, three for lateral) and contains the lesion (dumbbell‐shaped clip) and the iodine‐125 seed (arrow).

Lesion localisation was categorised as follows: SD 0–1 mm: optimal (Figs. 2 and 3), SD 1–5 mm: excellent, SD 5–10 mm: satisfactory and SD measurement > 10 mm: unsatisfactory (Fig. 4).

In cases in which the position of the seed or wire was deemed suboptimal by the performing radiologist, a second localisation using a wire was undertaken (Fig. 4).

An intra‐operative specimen X‐ray was taken in all cases to verify the removal of the target lesion and the seed or hookwire.

## Results

Patients were aged between 29 and 79 years, with an average of 57.9 years. The number undergoing prone stereotactic and upright tomosynthesis‐guided localisation and the localisation device used are listed in Table 1.

**Table 1:** Type of localisation and accuracy of the placement of the wire or seed.

Stereotactic localisation (*n* = 58)			Tomosynthesis localisation (*n* = 58)		
SD of wire/seed (mm)	Wires	Seeds	SD of wire/seed (mm)	Wires	Seeds
0–1	24	9	0–1	14	3
1–5	16	0	<5	13	10
5–10	5	1	5–10	6	6
>10	1	2	>10	4	2
Total	46	12	Total	37	21

For Stereo, shortest distance (SD) <5 mm in 86% wires and 75% of seeds; for Tomo, SD <5 mm in 72% wires and 62% of seeds.

Of 58 localisations performed using prone stereotactic guidance, a hookwire was used in 46 and a radioactive seed in 12. Optimal placement of the wire or seed was achieved in 33 of the 58 cases, with positioning considered excellent or satisfactory in further 22 cases, giving an acceptable positioning rate of 94.8% for conventional stereotactic guidance.

For the 58 patients in the upright tomosynthesis group, 37 cases were localised using a hookwire and 21 cases were localised using a radioactive seed. Optimal positioning was achieved in 17 of these cases, and excellent or satisfactory positioning in a further 35 cases, giving an acceptable positioning rate of 89.6%.

### Statistical analysis of results

The mean SD value for localisations performed using upright tomosynthesis (4.92) was found to be larger than for those undertaken using prone stereotactic guidance (2.26). In view of the non‐parametric nature of the data, the Mann–Whitney *U*‐test was used to test the level of significance of this difference. The resulting *P*‐value of 0.0005 is highly statistically significant.

### Technically unsatisfactory localisation procedures

There were three technically unsatisfactory localisations carried out under prone stereotactic guidance. In the case involving a hookwire, the thickened part of the wire was measured to be 15.1 mm deep to the lesion. A second wire was not deemed necessary, and the lesion was successfully excised. In the first seed case, the seed was located 12.7 mm short of the lesion. The radiologist inserted a wire from the same direction, which was placed 2.2 mm from the lesion. In the other case, the seed was 17.6 mm deep to the lesion. A further wire was inserted from the same direction lying 1.8 mm from the lesion.

There were six technically unsatisfactory localisations under upright tomosynthesis guidance, four hookwires and two seeds. For the unsatisfactory wire localisations, in the first case the wire was measured to be 10.8 mm short of the lesion and a second wire was accurately deployed also using tomosynthesis. In the second case, the initial wire, inserted using a craniocaudal approach, was extremely short of the lesion, at a distance of 32.5 mm. A second wire, inserted under stereotactic guidance and using the opposite approach of caudal cranial, was successfully placed at a distance of 4.3 mm from the lesion. The third case involved the wire being 19 mm deep to the lesion. After discussion with the surgeon, insertion of a further wire was not considered necessarily determined it was not necessary due to the position of the lesion and the planned surgery. In the fourth case, the wire was 13.7 mm short of the lesion, with the hook of the wire rather than the thickened portion adjacent to the targeted microcalcifications, and the surgeon did not require a second wire to be inserted.

**Figure 5:**
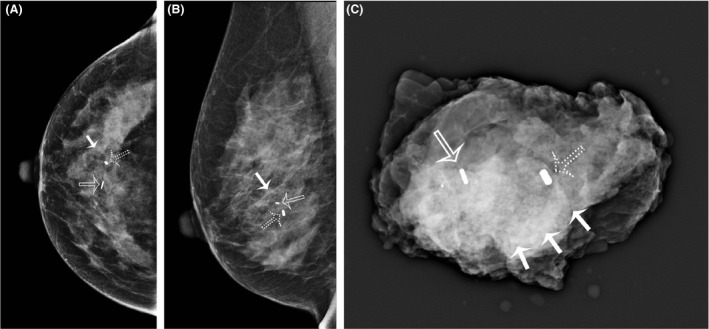
CC (A) and lateral (B) post‐procedural mammograms demonstrating the seed (hollow arrow) in relation to the residual calcifications (solid arrow) and the breast biopsy marker (dotted arrow). (C) Intra‐operative specimen X‐ray of the difficult localisation case, demonstrating the seed (hollow arrow), the residual calcifications (solid arrows) and the breast biopsy marker (dotted arrow) all within the specimen.

In the first case of a failed seed, the seed was measured to be 25.1 mm short, and a second wire was inserted under stereotactic guidance. In the final case, the seed was deployed 20.0 mm lateral to the target lesion of the residual calcification and 12.0 mm lateral to the breast biopsy marker deployed at the time of the biopsy in the CC view. On the lateral view, the seed was seen to be 5.0 mm inferior to the residual calcification (Fig. 4). This complicated case involved a great deal of discussion between the radiologist and the surgeon, with the result that a second localisation was not deemed necessary. A specimen radiograph demonstrates successful removal of the lesion, the seed and the breast biopsy marker (Fig. 5).

In five of the six cases of unsatisfactory pre‐operative localisation undertaken using upright tomosynthesis guidance, the lesion was noted to lie at or below the level of the nipple on the pre‐procedural lateral mammogram.

## Discussion

Tomosynthesis‐guided procedures, including pre‐operative lesion localisation, have been shown to be quicker and easier to perform than those using stereotactic methods,^9^, ^10^ with lower radiation dose.^10^ Stromal distortions and radial scars can be difficult to visualise using traditional stereotactic methods but are often clearly demonstrated with tomosynthesis.^9^ To date, no published studies have assessed the accuracy of lesion localisation with hookwires or seeds using tomosynthesis guidance. A study by Freer et al.^13^ has reported a 97% accuracy rate for tomosynthesis‐guided biopsy; however in this study, accuracy was judged by pathological concordance, not by the pre‐operative distance between the wire to the lesion.

The 2006 European guidelines for pre‐operative localisation of impalpable breast lesions using hookwires state that 95% of wires should lie within 10 mm of the lesion.^5^ This standard is considered generous, given the increasingly smaller size of mammographically detected breast cancers, and the dual aims of obtaining clear pathological margins and minimising unnecessary excision of normal tissue.

Mucci et al.^6^ proposed a new standard in which at least 90% of wires traverse the lesion in both planes. In the case of a substantially removed index lesion due to the previous diagnostic needle biopsy, where a marker clip becomes the surrogate target, however this requires clarification. We suggest that an SD measurement of <5 mm taken from the localisation target at the centre of the lesion or marker clip to the thickened segment of the wire or centre of a radioactive seed to be a reasonable standard.

Prone tables with tomosynthesis capabilities are now available to purchase and may alleviate some of the problems we discovered with performing pre‐operative lesion localisations upright under tomosynthesis guidance. However, it should be noted that the cost difference between an add‐on biopsy device for an existing upright mammography machine and a stand‐alone prone biopsy table is significant, and some centres may not have either the funds or the space available to choose between the two options.

### Factors influencing localisation accuracy

In five of the six technically unsatisfactory cases of upright tomosynthesis‐guided localisation, the lesion was seen to be at or below the level of the nipple on the lateral mammogram, with the approach chosen for localisation being craniocaudal from above. This highlights the importance of choosing the appropriate approach for insertion of the localising device, with the aim being to traverse the shortest distance through the breast wherever possible. This helps to minimise the movement of the wire or seed due to the accordion effect when the compression is released from the breast.^15^ As a direct result of this study, in our institution, localisation of a lesion at or below the nipple is now performed using a lateral approach unless the lesion is centrally located within the breast when the choice would be to locate the lesion stereotactically with the patient prone and using a CC from below approach.

An interesting observation from this study is that seeds placed using prone stereotactic guidance were either in the lesion itself or greater than 10 mm away. Conversely, seeds placed using upright tomosynthesis guidance were found to have a spread of acceptable locations, with most being situated within 5 mm of the target lesion, not actually in the lesion itself.

A possible explanation for this finding is the difference in the method used to determine the depth with each technique. With stereotactic guidance, accurate calculation of the depth of the lesion is dependent upon the radiologist selecting the same target on the pair of localisation images. The tip of the needle is then placed at this depth, and the seed is deployed into the centre of the lesion. If the same portion of the lesion has not been accurately selected on targeting, the depth measurement will be inaccurate, and the seed deployed outside the lesion. With tomosynthesis guidance, the deployment depth is judged by subtracting the lesion depth from the overall number of tomo slices. However, with tomosynthesis an additional five slices are reconstructed on the compression paddle side automatically in order to ensure the entire breast is imaged.^16^ These five slices are more likely to be significant in smaller breasts than in larger breasts. In a breast with a compressed thickness of 50 mm thick, 5 mm equates to 10% of the breast thickness; however, in a thinner breast with a compressed thickness of 20 mm, this additional 5 mm represents 25% of the total breast thickness. This could potentially affect the calculation of the location of the lesion.^16^ Breast biopsy markers are also visible across a number of tomographic slices. This can also result in slight miscalculations in depth when using a biopsy marker as the target for seed deployment.

Schrading et al.^10^ conducted a study comparing prone stereotactic breast biopsy with tomosynthesis biopsy and concluded that tomosynthesis biopsy was more accurate and took significantly less time to perform. While we do not have accurate data to confirm the time taken to perform each procedure during our study, it is our empirical experience that using upright tomosynthesis pre‐operative wire or seed guidance is a quicker procedure than prone stereotactic guidance, and this has been noted in the literature by other studies.^9^

### Level of experience of the radiologist

Five of the nine technically unacceptable localisations were performed by consultants, and the other four were performed by registrars or fellows. It is standard practice in our facility for the consultant to perform the localisation if it is likely to be complicated or difficult. Each of our five consultants had one failed localisation; however, three of the four failures performed by non‐consultants were performed by the same breast fellow. This suggests a possible problem with the technique used by this operator, with the introduction of bias into the results. If these cases are removed, the rates for acceptable localisation using stereotactic and tomosynthesis guidance are 96.5% and 93%, respectively.

### Study limitations

The limitations of our study include a small sample size and that we are reporting the experience of a single institution. Although retrospective in nature, the inclusion of a consecutive series of patients minimises potential selection bias.

## Conclusion

The degree of accuracy of pre‐operative localisation of impalpable breast lesions is higher with the use of prone stereotactic rather than upright tomosynthesis guidance. This was most evident with the placement of I‐125 seeds, and in cases where the target lesion was located below the level of the nipple.

## Conflicts of Interest

No authors have any disclosures or conflicts of interest.
